# The Amazonian Camu-Camu Fruit Modulates the Development of *Drosophila melanogaster* and the Neural Function of Adult Flies under Oxidative Stress Conditions

**DOI:** 10.3390/antiox13010102

**Published:** 2024-01-15

**Authors:** Elize Aparecida Santos Musachio, Rafaela Garay Pires, Eliana Jardim Fernandes, Stefani Andrade, Luana Barreto Meichtry, Dieniffer Espinosa Janner, Graziela Moro Meira, Euler Esteves Ribeiro, Fernanda Barbisan, Ivana Beatrice Mânica da Cruz, Marina Prigol

**Affiliations:** 1 Laboratory of Pharmacological and Toxicological Evaluations Applied to Bioactive Molecules (LaftamBio), Federal University of Pampa, Itaqui 97650-000, RS, Brazil; elizemusachio.aluno@unipampa.edu.br (E.A.S.M.); rafaelapires.aluno@unipampa.edu.br (R.G.P.); elianafernandes.aluno@unipampa.edu.br (E.J.F.); stefaniandrade.aluno@unipampa.edu.br (S.A.); luanameichtry.aluno@unipampa.edu.br (L.B.M.); dienifferjanner.aluno@unipampa.edu.br (D.E.J.); marinaprigol@unipampa.edu.br (M.P.); 2 Laboratory of Biogenomics, Federal University of Santa Maria, Santa Maria 97105-900, RS, Brazil; graziela.moro@acad.ufsm.br (G.M.M.); fernanda-barbisan.1@ufsm.br (F.B.); 3 Center for Research, Teaching and Technological Development-GERONTEC, Open University Foundation for the Elderly, Manaus 69029-040, AM, Brazil; unatieuler@gmail.com; 4 Graduate Program in Gerontology, Federal University of Santa Maria, Santa Maria 97105-900, RS, Brazil; 5 Graduate Program in Pharmacology, Federal University of Santa Maria, Santa Maria 97105-900, RS, Brazil

**Keywords:** camu-camu, *Drosophila melanogaster*, larvae, oxidative stress

## Abstract

Camu-camu (*Myrciaria dubia*) is known for its antioxidant properties, although little is known about its developmental safety effects, particularly on adult neural function under basal redox and oxidative stress conditions. Therefore, this study sought to address this gap by conducting three complementary protocols using *Drosophila melanogaster* to investigate these effects. The initial assays revealed that second-stage larvae consumed diets supplemented with various concentrations of camu-camu uniformly, establishing a 50% lethal concentration at 4.799 mg/mL. Hence, non-lethal (0.1, 0.5, and 1 mg/mL) and sub-lethal (5 and 10 mg/mL) concentrations were then chosen to evaluate the effects of camu-camu on preimaginal development and adult neural function. Our observations showed that camu-camu impacts the expression of antioxidant enzymes, reactive species, and lipoperoxidation. Notably, sub-lethal concentrations decreased preimaginal viability and locomotor activity, negatively influenced geotaxis and acetylcholinesterase activity, and increased reactive species, catalase, and glutathione S-transferase activity in flies. Additionally, the protective effects of camu-camu against oxidative stress induced by iron (20 mM) were assessed. Flies supplemented with 0.5 mg/mL of camu-camu during the larval period showed improved neural viability and function, and this supplementation was found to protect against oxidative stress. These findings are instrumental in evaluating the safety and efficacy of commercial supplements based on camu-camu, offering significant insights for future research and application.

## 1. Introduction

Consistent epidemiological and experimental data have shown that plant-based diets may be related to antioxidant and anti-inflammatory mechanisms associated with the matrix of phytochemicals found in fruits, vegetables, herbs, and spices [1]. The Amazon is known worldwide for its rich biodiversity of fruit species with promising biological activities [2]. Native fruits have been used empirically by the Amazonian populations (e.g., indigenous and riverside Amazonian peoples) as a source of nutrients and to treat various diseases. This has garnered the attention of the scientific community to investigate the mechanisms involved in these beneficial effects [2,3], particularly since the therapeutic properties of Amazonian fruits have been shown to lay the groundwork for bioprospecting new drugs or functional supplements [4,5].

Among the various Amazonian fruits, camu-camu (*Myrciaria dubia*)stands out. It is a rich source of potassium, iron, calcium, and phosphorus and has a high concentration of polyphenols, carotenoids, and anthocyanins. Most notably, it has the highest concentration of vitamin C of any fruit in Brazil [6]. However, the consumption of fresh camu-camu is limited by its bitter taste, and the commercialization of the fruit is hindered by its perishability due to its high water content, which makes it more susceptible to chemical and enzymatic degradation, resulting in losses in nutritional value and shelf life [2,5]. Therefore, pasteurized berries are used to produce camu-camu powder, allowing easier transport for export and practical use in the food industry [2,7].

Evidence has suggested that camu-camu powder could be a relevant antioxidant supplement due to its reducing properties, which help neutralize free radicals and chelate transition metals, thereby acting in both the initiation and propagation phases of the oxidative process [6]. However, while using powdered camu-camu as a food supplement is a reality, there is a lack of established safe dosages, not only considering its potential toxic effect but also its influence on different stages of development through the differential modulation of oxidative metabolism. Studying the effects of antioxidants on development, especially their potential impact on adult organisms, is challenging and complex in vertebrate models or humans, making the fruit fly *Drosophila melanogaster* a viable alternative. Due to their enzymatic and non-enzymatic antioxidant systems, which are similar to those in mammals, these flies provide an efficient model for assessing the toxicity of substances related to oxidative stress [8]. The fruit fly model, particularly under conditions of iron-induced oxidative stress—a well-established scenario in the scientific literature [9]—has been instrumental in evaluating the effects of various antioxidant molecules. Additionally, *D. melanogaster* has been widely utilized in researching neurodegenerative conditions [1,2]. Thus, this organism offers a valuable means to investigate the influence of camu-camu powder on neurodevelopment and its subsequent impact on adult neural function, both in the presence and absence of pro-oxidant agents such as iron.

To determine the concentration range of dietary supplementation with camu-camu that is not toxic to *D. melanogaster* larvae, this study began by assessing pupa formation and viability and adult *D. melanogaster* emergence. We then evaluated the impact of camu-camu supplementation during the preimaginal period on the neural function and modulation of oxidative markers in adult fruit flies with and without oxidative stress induced by iron exposure.

## 2. Materials and Methods

### 2.1. Camu-Camu Powder

Organic camu-camu powder was purchased from Nativas Organics. The powder was made from ripe camu-camu berries, which were washed and gently dehydrated at low temperatures to preserve the nutrients. The dehydrated berries were then ground to a powder. Table 1 describes the nutrients in camu-camu powder as provided by the manufacturer.

### 2.2. D. melanogaster Stock

*D. melanogaster* flies (Harwich strain) were reared and fed in a controlled temperature environment (25 ± 1 °C) of 60–70% humidity and 12h light and 12h dark cycle. The flies were cultured on a standard laboratory diet composed of corn flour (76.59%), wheat germ (8.51%), sugar (7.23%), powdered milk (7.23%), salt (0.43%), and methylparaben. The first two experimental protocols were conducted using second-stage larvae.

### 2.3. Experimental Design

**Figure 1:**
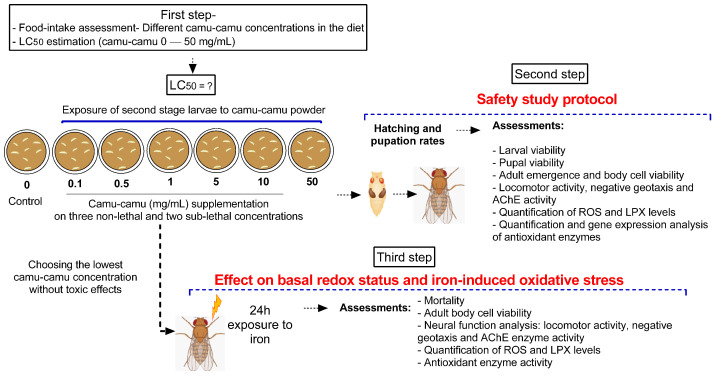
Schematic representation of the experimental design.

Second-stage larvae were transferred from their breeding grounds to plates containing the diet mixed with camu-camu powder, with each plate hosting 50 larvae for every experimental group. The predetermined amounts of camu-camu powder, measured in milligrams, were incorporated into 10 mL of a standard, ready-made diet with a pasty consistency prepared at room temperature. Ten independent experiments were conducted to ensure comprehensive analysis and behavioral testing. The experimental design was structured in three stages. The first stage focused on assessing the safety of various doses of camu-camu powder on *D. melanogaster* during the larval stage. The second stage evaluated the impact of camu-camu supplementation during the preimaginal period on the development and neural function of adult flies. In the third and final stage, we explored the effects of camu-camu powder supplementation throughout the preimaginal period on both the basal redox status and the oxidative stress response induced by exposure to 20 mM of iron (Fe) in adult flies. Figure 1 illustrates the experimental protocols employed.

### 2.4. Step One: Camu-Camu Powder Safety Assessment

The initial phase involved conducting a food consumption test to ensure the larvae were consuming the camu-camu powder. This was imperative for determining the lethal concentration of second-stage larvae. With these results, various concentrations of camu-camu were selected to observe their effects on the development of *D. melanogaster*, focusing particularly on pupation rates and emergence. The larval stage is notably sensitive to the toxic effects of substances since it is a critical period for the formation of antioxidant defenses. Therefore, assessing the potential toxicity of camu-camu during this stage was essential [10,11]. Post-hatching, the study also involved analyzing markers of oxidative stress and the behavior of the adult flies.

#### 2.4.1. Food Intake

To evaluate the extent of the larvae’s consumption of the camu-camu powder, a food intake test was performed according to the literature [10]. In this test, ten larvae from each group were placed in Petri dishes containing the diet mixed with different concentrations of camu-camu powder, each supplemented with 0.5% of FD&C Blue No. 1 dye (Brilliant Blue FCF). The larvae were allowed to feed for 1 h, after which they were cleaned and homogenized in KPi buffer (50 mM, pH 7.5). This homogenate was then centrifuged at 1800× *g* for 5 min, and the supernatant was measured for absorbance at 629 nm using a microplate reader. The test was replicated in 5 independent experiments, with samples analyzed in duplicate. Results were reported as percentages relative to the control group.

#### 2.4.2. Lethal Concentrations

The LC_50_ (lethal concentration for 50% of the population) was determined following the protocol established by Musachio et al. [12]. For this purpose, second-stage larvae of *D. melanogaster* were placed in Petri dishes under various conditions: (1) a control group (standard diet only) and groups with different concentrations of camu-camu mixed with the standard diet, including (2) 0.1 mg/mL, (3) 0.5 mg/mL, (4) 1 mg/mL, (5) 5 mg/mL, (6) 10 mg/mL, and (7) 50 mg/mL. Each plate hosted 50 larvae. The larvae were exposed to these treatments for 48 h, after which mortality was recorded to calculate the LC_50_. This procedure was repeated in six separate experiments (n = 6).

#### 2.4.3. Determination of Safe Concentrations of Camu-Camu Powder

Six experimental groups were set up to determine the safe concentrations of camu-camu powder. Fifty second-stage larvae were placed in Petri dishes containing 10 mL of the diet with respective treatments. These included three non-lethal and two sub-lethal concentrations of camu-camu, determined based on the LC_50_ calculation. When the larvae reached the pupal stage, they were transferred to flasks containing a standard diet, where they remained until hatching. This ensured that exposure to camu-camu was limited to the larval developmental period. Post-hatching, these flies entered the adult phase of *D. melanogaster* and were subjected to behavioral and biochemical tests.

#### 2.4.4. Pupation and Hatching Rate

Fifty second-stage larvae were placed in each diet treatment and monitored until they reached the pupal stage. They were transferred to flasks with a standard diet during the pupation process and remained there until emergence. The number of larvae that successfully transitioned to the pupal stage was counted. Afterward, the number of adult flies that emerged was recorded based on the total number of pupae formed.

### 2.5. Iron-Induced Oxidative Stress Model

This part of the study evaluated the enduring antioxidant protective effects of preimaginal camu-camu supplementation in adult flies exposed to iron (Fe). We utilized the iron-induced oxidative stress model in *D. melanogaster*, as outlined by Poetini et al. [13]. Iron was administered as Fe II sulfate (FeSO_4_) at a concentration of 20 mM dissolved in a 1% sucrose solution. Given the safety results obtained in the first stage, a concentration of 0.5 mg/mL of camu-camu was chosen for the induced model. Four experimental groups were established: (1) control (flies not exposed to camu-camu during the larval period), (2) camu-camu (CC) (flies exposed to camu-camu during the larval period), (3) Fe (flies not exposed to camu-camu during the larval period but subjected to Fe in adulthood), and (4) camu-camu + Fe (CC + Fe) (flies exposed to camu-camu as larvae and to Fe in adulthood). In groups (3) Fe and (4) CC + Fe, adult flies (24 h old) were transferred to a flask containing filter paper soaked in 20 mM iron sulfate mixed with 1% sucrose. For the (1) control and (2) CC groups, adult flies (24 h old) were placed in flasks with filter paper soaked in 1% sucrose. After 24 h of exposure to their respective treatments, the flies underwent behavioral tests and biochemical parameter analysis.

### 2.6. Adult Neural Function Assays

#### 2.6.1. Locomotor Activity Behavior during an Open-Field Test

The open-field behavioral tests were conducted according to the methods described by Connolly [14] and adapted by Musachio et al. [15]. A total of 60 flies per group were utilized, with 15 flies from each group participating in four independent experiments. Before testing, flies were cryoanesthetized for 30 s at −2 °C, then placed individually in transparent polycarbonate Petri dishes (9 mm in diameter) with 1 cm^2^ quadrants marked on the lid. Following a 2 min acclimation period post-anesthesia, the test commenced. This test assesses the spontaneous locomotor activity of the flies by visually observing the number of quadrants traversed within 60 s from the start of the timer. The test was performed in duplicate, and mean values were computed.

#### 2.6.2. Negative Geotaxis Behavior Assay

The negative geotaxis test, as outlined by Jimenez-Del-Rio et al. [16], was conducted with five flies from each group across four independent trials. Flies were placed individually in vertical cylindrical tubes for acclimatization over 2 min. The test commenced with a light tap at the base of the tube, simultaneously starting the stopwatch. Each fly had a maximum of 2 min to climb to a height of 8 cm within the tube. This climbing process was repeated five times for each fly, with a 3 min interval between each attempt. The average time taken across the five attempts by the five flies (for each group) was used for statistical analysis.

### 2.7. Adult D. melanogaster Biochemical and Molecular Assays

The flies were transferred from their treatment containers into tubes and subsequently euthanized at a low temperature (−80 °C for 1 h). To evaluate enzyme activity (SOD, CAT, GST, and AChE), ten whole flies were homogenized in 400 µL of ice-cold HEPES buffer. The homogenate was centrifuged at 1000× *g* for 10 min, the pellet was discarded, and the supernatant was used for further analysis.

#### 2.7.1. *D. melanogaster* (Body) Cell Viability Assay

Cell viability in *D. melanogaster* was assessed according to Franco and collaborators [17], with some modifications. Fifteen flies per group were homogenized in 400 µL of Tris buffer (20 mM, pH 7.0). The homogenate was then centrifuged at 1000× *g* for 10 min. To each microplate well, a mixture of 20 µL of the sample, 180 µL of 20 mM Tris buffer (pH 7.0), and 10 µL of resazurin were added and incubated for 2 h in the dark at room temperature. The reduction of resazurin to resorufin, which can be quantitatively measured at 574 nm, was utilized to assess metabolic viability. The absorbance results were expressed as a percentage relative to the control group.

#### 2.7.2. Acetylcholinesterase Activity Assay

Acetylcholine, a neurotransmitter crucial for cognition, attention, and arousal functions, is prevalent in the nervous systems of vertebrates and some invertebrates [18]. It is catalyzed by acetylcholinesterase (AChE), and elevated AChE activity has been linked to various neural dysfunctions and neurodegenerative diseases, including Alzheimer’s. In *D. melanogaster*, exposure to Fe has been shown to increase AChE levels [19]. Our study quantified AChE in adult fruit flies reared in preimaginal stages in camu-camu-supplemented cultures using a modified method from the literature [20]. A reaction mixture consisting of 0.25 M KPi buffer (pH 8.0) and 5 mM DTNB (5, $5^{\prime }$ -dithiobis 2-nitrobenzoic acid) was prepared. At the time of analysis, 935 µL of this mixture, 50 µL of acetylthiocholine (AcSCh) (7.25 µM), and 50 µL of the supernatant from sample preparation were added to a cuvette. AChE activity was measured spectrophotometrically at 412 nm over 2 min and expressed as µmol AcSCh/h/mg protein.

#### 2.7.3. Reactive Species Quantification

Reactive species (RS) was quantified using the method described by Pérez- Severiano et al. [21], which allows for measuring both reactive oxygen and nitrogen species. Twenty larvae were homogenized in 1000 µL of Tris buffer (10 mM, pH 7.0). Post-centrifugation at 1000× *g* for 5 min, 34 µL of the supernatant was mixed with 964 µL of HEPES buffer (pH 7.0) and 10 µL of DCFH-DA (2′7′ dihydrodichlorofluorescein diacetate) in test tubes. The samples were then incubated at 37 °C for 1 h. During this period, DCFH-DA is oxidized by RS in the samples to form dichlorofluorescein, a highly fluorescent metabolite. The fluorescence intensity of dichlorofluorescein, which is proportional to the RS amount, was measured using a fluorimeter (488 nm excitation and 520 nm emission). This analysis was performed in duplicate across three independent experiments, with results expressed relative to the control.

#### 2.7.4. Lipid Peroxidation Quantification

The lipid peroxidation levels were determined using the method of thiobarbituric acid reactive species (TBARS) as described by Ohkawa et al. [22] with adaptations. First, ten flies per group were weighed and then homogenized in 400 µL of HEPES buffer. The homogenate was centrifuged at 1000× *g* for 10 min. Of the resulting supernatant, 100 µL was transferred to test tubes containing the reaction mixture: 125 µL of thiobarbituric acid (8%), 12 µL of acetic acid, 50 µL of sodium dodecyl sulfate (1.2%), and 25 µL of distilled water. The samples were then placed in a water bath at 95 °C for 2 h, and the absorbance was measured at 532 nm using a microplate reader. Results were expressed as nmol MDA/mg tissue.

#### 2.7.5. Superoxide Dismutase Activity Quantification

Superoxide dismutase (SOD) activity was evaluated based on the method by Pinheiro et al. [23] with modifications, which involved monitoring the inhibitory effect of SOD on quercetin oxidation. Thirty whole flies were homogenized in 3000 µL of HEPES buffer, and the homogenate was centrifuged at 14,000× *g* for 10 min. For the assay, 10 µL of the supernatant was diluted in 90 µL of HEPES buffer. To this, 1 mL of reaction mixture (0.025 M sodium phosphate buffer with 0.1 mM EDTA and TEMED), 10 µL of sample, and 50 µL of quercetin were added sequentially into a cuvette. The absorbance was read at 406 nm over 2 min, corrected for the protein concentration in the sample, and the percentage inhibition of quercetin oxidation was calculated. This assay was performed in five independent experiments, with samples analyzed in duplicate and the results expressed as SOD units per mg protein (U/mg protein).

#### 2.7.6. Catalase Activity Quantification

Catalase (CAT) activity was measured following the method by Aebi [24], as adapted by Jimenez-Del-Rio et al. [16]. The assay assessed the enzyme’s capacity to degrade H_2_O_2_. From the same supernatant used for SOD analysis, a 30 µL aliquot was transferred to a quartz cuvette, followed by the addition of 2000 µL of reaction mixture (0.25 M KPi buffer/2.5 mM EDTA pH 7.0, Triton, and H_2_O_2_). Absorbance was recorded at 412 nm for 2 min. The assay was conducted in duplicate across five independent experiments. Results were adjusted for the protein concentration of the sample and expressed as CAT units per mg protein (U/mg protein).

#### 2.7.7. Glutathione S-Transferase Activity Quantification

The detoxifying enzyme glutathione S-transferase (GST) was determined following the method described by Santos Musachio et al. [25]. In this analysis, GST catalyzes the conjugation of 1-chloro-2,4-dinitrobenzene with reduced glutathione (GSH), producing the thioether 4-dinitrophenyl glutathione. For this analysis, samples were prepared from the supernatant used to evaluate SOD and CAT activities. In an acrylic cuvette, 30 µL of supernatant was added to 1000 µL of a solution containing 0.25 M KPi buffer, 2.5 mM EDTA, 100 mM GSH, and distilled water. The reaction was initiated by adding 20 µL of 50 mM 1-chloro-2,4-dinitrobenzene (substrate). The analysis was performed in 5 independent experiments with duplicate samples, and the results were corrected for protein concentration. The corrected results were expressed as nanomoles of enzyme activity per milligram of protein (nmol/mg protein).

#### 2.7.8. Total Protein Quantification

Enzyme activity values were corrected for total protein concentration in the samples and quantified using Bradford’s method [26]. This technique employs Coomassie brilliant blue BG-250 dye, which interacts with protein components containing basic or aromatic amino acids. This interaction alters the dye’s equilibrium towards its anionic form, absorbing strongly at 595 nm.

### 2.8. Gene Expression by qRT-PCR Molecular Assay

Post-hatching, 30 flies from each treatment group were macerated, and total RNA was extracted using Trizol Reagent (Ludwig, Alvorada, RS, Brazil) as per the manufacturer’s instructions. RNA quantity and purity were assessed using a NanoDrop spectrophotometer (NanoDrop Technologies, Wilmington, DE, USA). Reverse transcription involved incubating 1 µg/mL RNA with 0.2 µL DNAase (Invitrogen Life Technologies, Waltham, MA, USA) at 37 °C for 5 min, followed by a 10 min incubation at 65 °C. cDNA synthesis was carried out using 1 µL Iscript cDNA and 4 µL Mix Iscript (Bio-Rad Laboratories, Hercules, CA, USA). The process included heating at 25 °C for 5 min, 42 °C for 30 min, and 85 °C for 5 min, followed by a 60 min incubation at 5 °C. After obtaining the cDNA, qRT-PCR was performed as previously described by da Cruz et al [27]. For each sample, qRT-PCR was performed in triplicate using 1 µM of each primer, 1000 ng/µL of cDNA, RNase-free water, and 2 X QuantiFast SYBR Green PCR Master Mix (Qiagen Biotechnology, Hilden, NW, Germany) in a final reaction volume of 20 µL. The qRT-PCR cycle in the Rotor Gene thermocycler (Qiagen Biotechnology, Hilden, NW, Germany) consisted of 95 °C for 3 min, 40 cycles of 95 °C for 10 s, and 60 °C for 30 s, and a melting curve from 60 to 90 °C with 0.5 °C increments for 5 s. For each sample, qRT-PCR was performed in triplicate. The GPDH gene was used as the housekeeping gene for normalization. Expression values < 1 indicated gene downregulation, while values > 1 indicated upregulation compared to the control. Primer pairs were designed from GenBank sequences (Table 2) and synthesized by integrated DNA Technologies, Leuven, Belgium.

### 2.9. Statistical Analysis

The data were analyzed using GraphPad Prism software version 8. One- or two-way ANOVA was employed, with the Shapiro–Wilk test assessing data normality. Tukey’s test was applied for normally distributed data, and the results were presented in bar graphs showing the mean ± standard error of the mean (SEM). Non-normally distributed data were analyzed using Dunn’s test, with results depicted in box-and-whisker plots illustrating the median, interquartile range, and minimum and maximum values. The LC_50_ was determined using linear regression analysis with a 95% confidence interval. Statistical significance was defined as *p* < 0.05.

## 3. Results

### 3.1. Safety Evaluation of Different Concentrations of Camu-Camu Powder

#### 3.1.1. Food Intake Test and LC_50_

**Figure 2:**
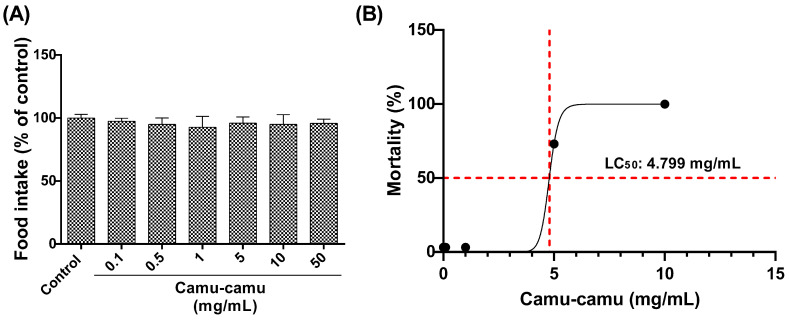
Food consumption by larvae exposed to diets supplemented with different concentrations of camu-camu powder (**A**) and evaluation of the lethal concentration of camu-camu powder that killed 50% (LC_50_) of exposed larvae (**B**). For 48 h, second-stage *D. melanogaster* larvae were exposed to different concentrations of camu-camu powder (0.1, 0.5, 1, 5, 10, and 50 mg/mL).

The larvae’s consumption of diets with varying concentrations of camu-camu (0.1, 0.5, 1, 5, 10, and 50 mg/mL) was evaluated (Figure 2A). No significant differences were observed among the groups, indicating uniform acceptance of the diets by the second-stage larvae. The LC_50_ for larvae exposed to these camu-camu concentrations was then determined as 4.799 mg/mL after 48 h of exposure (Figure 2B).

#### 3.1.2. Hatching and Pupation Rate

**Figure 3:**
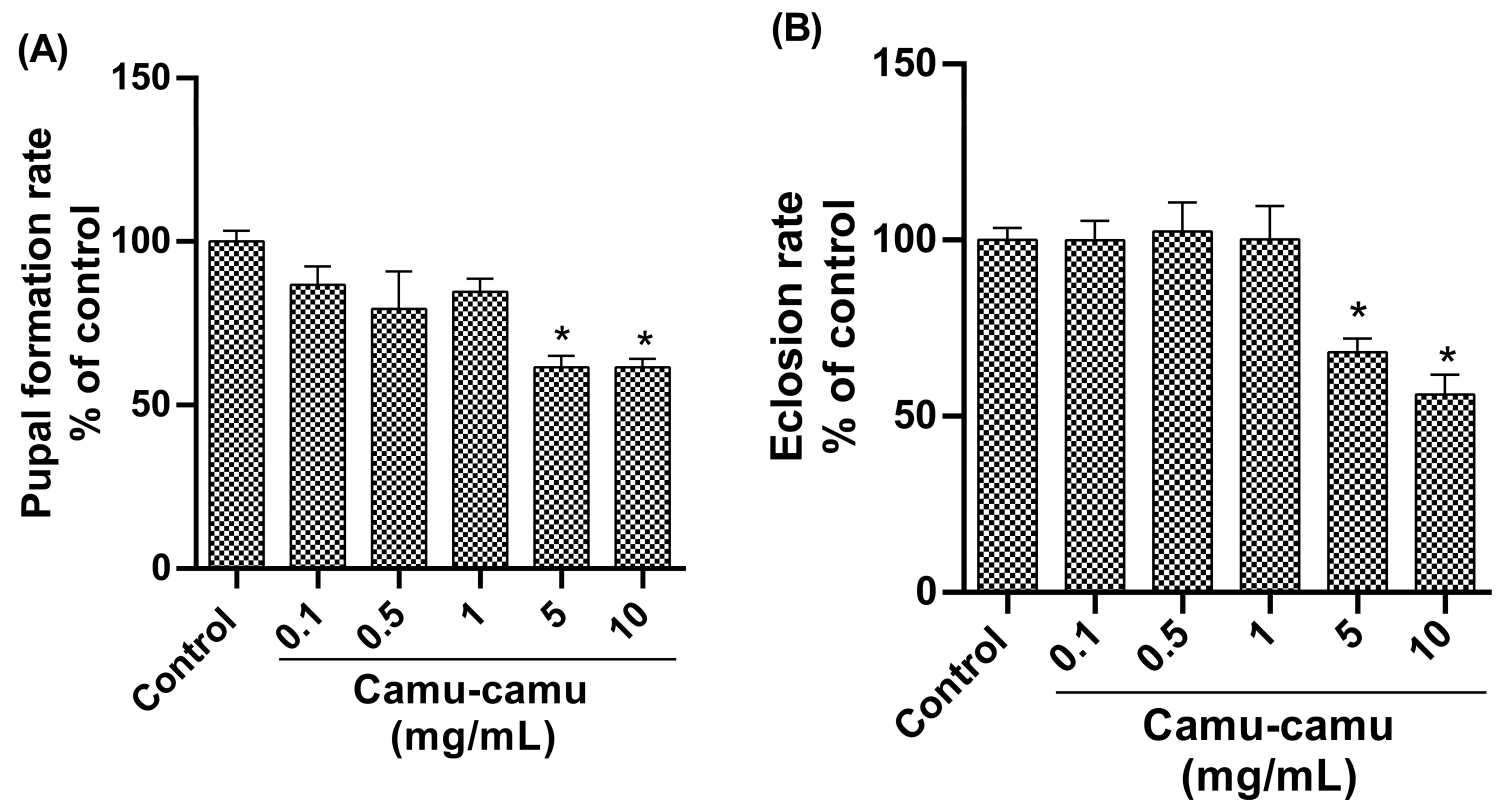
Pupation (**A**) and hatching (**B**) rates of *D. melanogaster* exposed to different concentrations of camu-camu powder. The graphs compare the different concentrations of camu-camu to the control group. * Statistically significant difference (*p* < 0.05). Values were expressed as mean ± standard error of the mean (SEM).

Analysis of the pupation rate (Figure 3A) revealed a significant reduction in pupae formation in the groups exposed to 5 and 10 mg/mL camu-camu (*p* = 0.0008 and *p* = 0.0190, respectively) compared to the control. Similarly, the hatching rate was lower in these high-concentration groups (Figure 3B), indicating impaired pupal development at 5 and 10 mg/mL of camu-camu (*p* = 0.0190 and *p* = 0.0008, respectively).

#### 3.1.3. Behavioral Tests and AChE Activity

**Figure 4:**
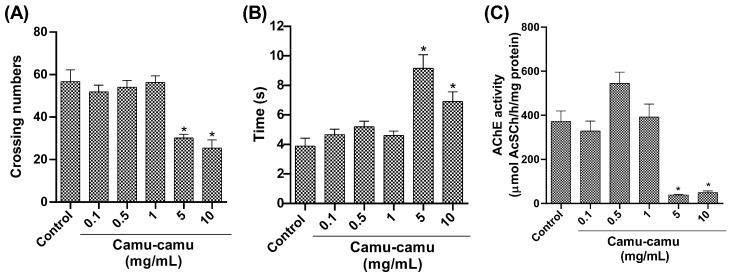
Behavior of *D. melanogaster* exposed to different camu-camu concentrations during development. (**A**) Open-field test, (**B**) negative geotaxis, and (**C**) acetylcholinesterase (AChE) activity. The graphs compare different camu-camu concentrations to the control group. * Statistically significant difference (*p* < 0.05). Values were expressed as mean ± standard error of the mean (SEM).

In the open-field test (Figure 4A), locomotor activity decreased at the highest camu-camu concentrations (5 and 10 mg/mL; *p* = 0.0346 and *p* = 0.0038, respectively). The negative geotaxis test (Figure 4B) showed increased climbing time at these concentrations, significantly differing from the control (*p* = 0.0346 and *p* = 0.0038). AChE activity also significantly decreased at high camu-camu concentrations (5 and 10 mg/mL) compared to the control group (*p* = 0.0011 and *p* = 0.0014, respectively) (Figure 4C).

#### 3.1.4. Quantification of Reactive Species, Lipid Peroxidation Levels, and Cell Viability

**Figure 5:**
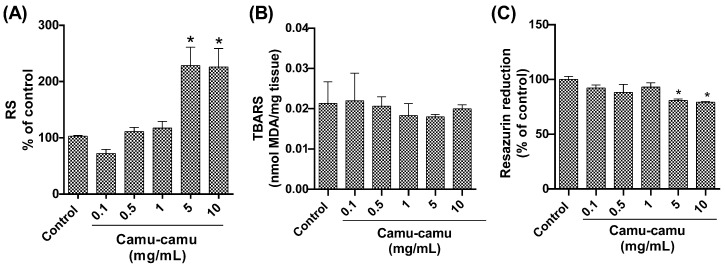
Behavior of *D. melanogaster* exposed to different camu-camu concentrations during development. (**A**) Reactive species, (**B**) lipid peroxidation by quantification of thiobarbituric acid reactive species (TBARS), (**C**) evaluation of cell viability by the resazurin reduction method. The graphs compare different concentrations of camu-camu to the control group. * Statistically significant difference (*p* < 0.05). Values were expressed as mean ± standard error of the mean (SEM).

Quantitative analysis of reactive species (Figure 5A) revealed an elevated level in Drosophila groups subjected to the highest camu-camu concentrations (5 and 10 mg/mL), with significant increases (*p* = 0.0014 and *p* = 0.0016, respectively) compared to controls. Conversely, lipid peroxidation levels, assessed via TBARS analysis, did not exhibit significant differences from the control group (Figure 5B). Regarding cell viability, as measured by the resazurin assay (Figure 5C), a noticeable decline in metabolic capacity was observed in the fly groups exposed to 5 and 10 mg/mL during development, compared to the control (*p* = 0.0363 and *p* = 0.0220, respectively).

#### 3.1.5. Antioxidant and Detoxification Enzyme Activity

**Figure 6:**
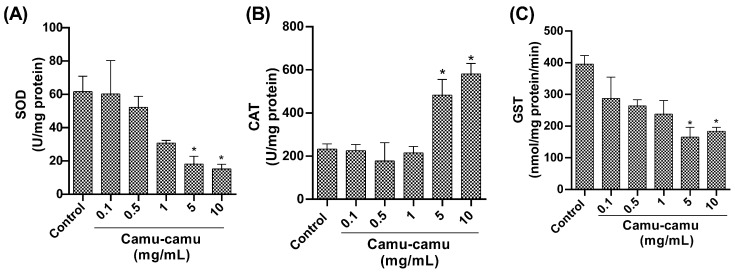
Evaluation of (**A**) superoxide dismutase (SOD), (**B**) catalase (CAT), and (**C**) glutathione-S-transferase (GST) activity in *D. melanogaster* exposed to different concentrations of camu-camu powder. The graphs compare the different concentrations of camu-camu to the control group. * Statistically significant difference (*p* < 0.05). Values are presented as mean ± standard error of the mean (SEM).

Regarding antioxidant enzymes, our study found a notable reduction in SOD activity (Figure 6A) in flies exposed to 5 and 10 mg/mL camu-camu concentrations during development compared to the control group (*p* = 0.0472 and *p* = 0.0272, respectively). Additionally, we observed an increase in CAT activity (Figure 6B) at 5 and 10 mg/mL (*p* = 0.0382 and *p* = 0.026, respectively) compared to the control. Furthermore, we found a significant reduction in GST activity (Figure 6C) at 5 and 10 mg/mL compared to the control (*p* = 0.050 and *p* = 0.0105, respectively).

#### 3.1.6. SOD and CAT Expression

**Figure 7:**
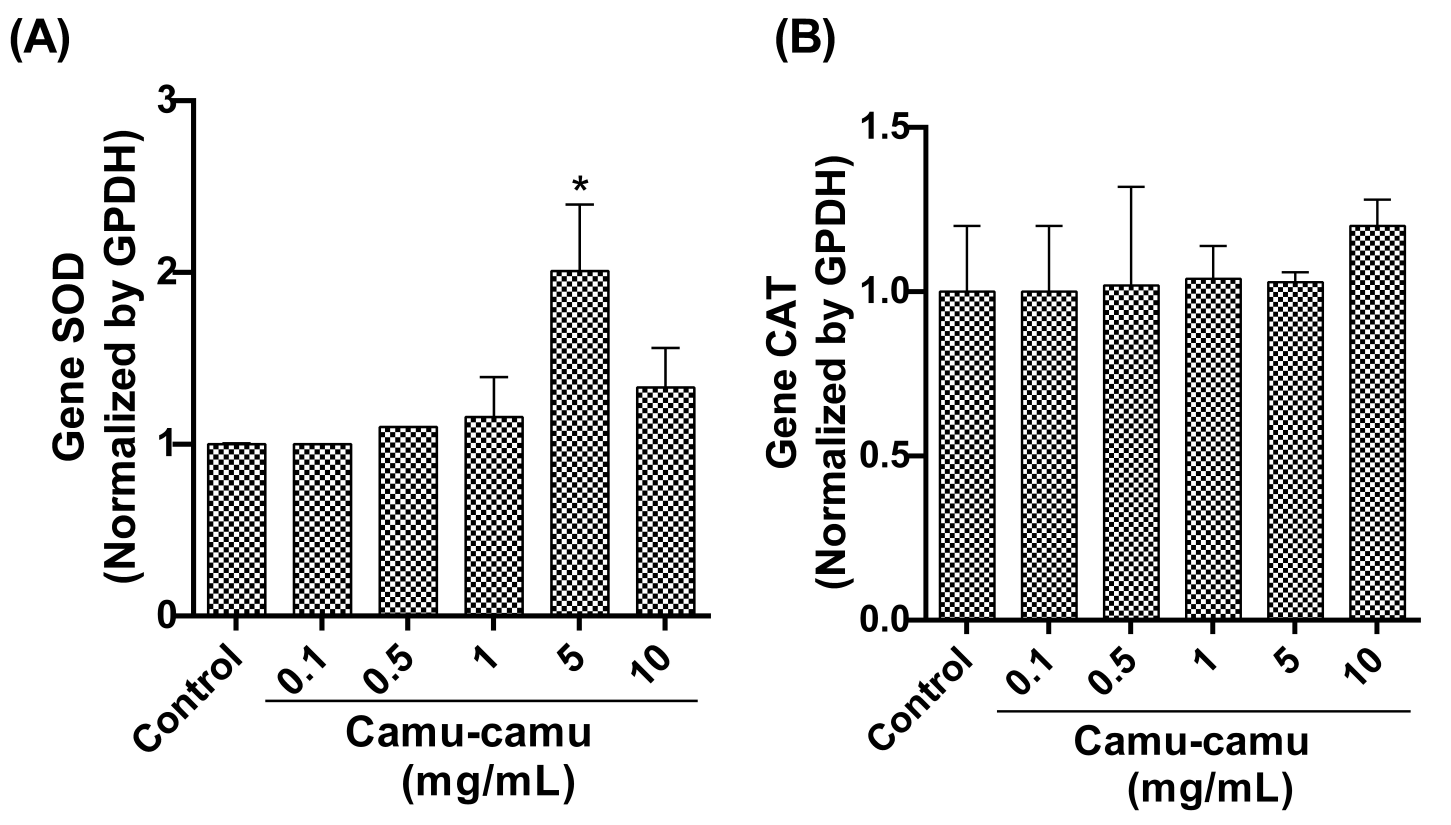
Evaluation of gene expression by analyzing and quantifying (**A**) superoxide dismutase (SOD) and (**B**) catalase (CAT) in *D. melanogaster* larvae exposed to varying concentrations of camu-camu powder during development. The graphs compare these concentrations to the control group. * Statistically significant difference (*p* < 0.05). Values are presented as mean ± standard error of the mean (SEM).

Our qRT-PCR analysis focused on the expression of detoxification antioxidant enzyme genes revealed a significant upregulation in SOD gene expression (Figure 7A) in flies exposed to a 5 mg/mL camu-camu concentration compared to the control (*p* < 0.0001). However, CAT gene expression (Figure 7B) showed no statistically significant difference between the exposed and control groups.

### 3.2. The Protective Effect of Camu-Camu Powder against Fe-Induced Oxidative Stress

To investigate the protective potential of camu-camu powder against Fe-induced oxidative stress, a concentration of 0.5 mg/mL was selected. This chosen concentration showed no adverse effects in a basal redox state and is approximately ten times lower than the LC_50_ concentration. In our graphical representations, CC denotes the flies exposed to 0.5 mg/mL CC powder during larval development, Fe represents flies not exposed to CC during development but were subjected to Fe for 24 h post-hatching. The CC + Fe group includes flies exposed to 0.5 mg/mL CC during development, followed by 24 h of Fe exposure upon hatching.

#### 3.2.1. Locomotor and Climbing Capacity and AChE Activity in Relation to Fe Exposure

**Figure 8:**
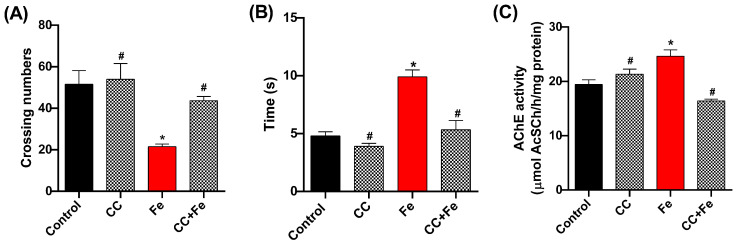
Behavioral tests and acetylcholinesterase (AChE) activity of *D. melanogaster* exposed to camu-camu (CC) during development and iron (Fe) as adults. Behavioral tests included (**A**) open-field, (**B**) negative geotaxis, and (**C**) AChE activity. * Statistically significant difference (*p* < 0.05); # statistically significant difference from the Fe group. Values were expressed as mean ± standard error of the mean (SEM).

In the behavioral assessments, the open-field test (Figure 8A) revealed a decrease in locomotion in the Fe-only group compared to controls (*p* = 0.0202). However, both the CC (0.5 mg/mL) and CC + Fe groups displayed enhanced locomotor abilities relative to the Fe-only group (*p* = 0.0047 and *p* = 0.0441, respectively). In the negative geotaxis test (Figure 8B), Fe-exposed flies showed delayed climbing times in comparison to the control group (*p* = 0.0008). Notably, flies in the CC + Fe group demonstrated no impairment in climbing ability, ascending 8 cm in a shorter duration than the Fe group (*p* = 0.0016). As for AChE activity (Figure 8C), the Fe-only group exhibited a decrease in enzyme activity compared to the control group (*p* = 0.0125). Conversely, the CC and CC + Fe groups maintained AChE activity at levels comparable to the control. Moreover, their activity was notably higher than that of the Fe-only group (*p* = 0.0166 and *p* = 0.0007, respectively).

#### 3.2.2. Oxidative Stress Markers, Cell Viability, and Mortality of Flies in Relation to Fe Exposure

**Figure 9:**
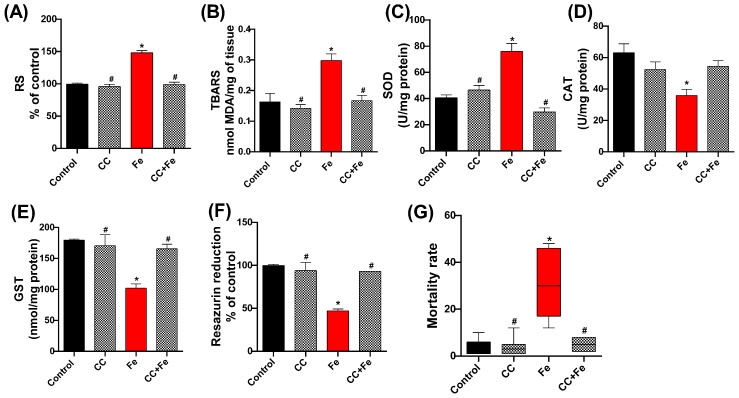
Quantification of (**A**) reactive species (RS), (**B**) lipid peroxidation via thiobarbituric acid reactive species (TBARS), and evaluation of enzyme activity: (**C**) superoxide dismutase (SOD), (**D**) catalase (CAT), and (**E**) glutathione-S-transferase (GST), (**F**) cell viability by the resazurin reduction method, and (**G**) mortality of *D. melanogaster* exposed during development to camu-camu (CC) versus 24 h of exposure to iron (Fe) as adults. Values were expressed as mean ± standard error of the mean (SEM). The graph representing mortality (**G**) showed a non-Gaussian distribution; therefore, a non-parametric test was applied. Plots show the median, interquartile range, and minimum and maximum values. Values are expressed as median and range (interquartile interval). * Statistically significant difference (*p* < 0.05) compared to the control group; # statistically significant difference from the Fe group (CC-exposed flies).

In the assessment of oxidative stress markers, RS levels in the Fe group were significantly higher than the control (*p* < 0.0001) (Figure 9A). However, both the CC and CC + Fe groups exhibited RS levels akin to the control, suggesting reduced RS production compared to the Fe group (*p* < 0.0001 for both). For lipid peroxidation (Figure 9B), an elevated TBARS level was observed in the Fe-only group relative to controls (*p* = 0.0077). In contrast, flies in the CC and CC + Fe groups maintained TBARS levels at control values, indicating lower lipid peroxidation than the Fe group (*p* = 0.0032 and *p* = 0.0090, respectively).

In terms of enzymatic activity, SOD (Figure 9C) showed increased activity in the Fe-only group compared to controls (*p* < 0.0001). The CC and CC + Fe groups, however, maintained SOD activity at control levels, signifying reduced activity relative to the Fe group (*p* < 0.0001 for both). Analysis of CAT activity (Figure 9D) revealed a decrease in enzyme activity in flies exposed solely to Fe compared to the control group (*p* = 0.0129). Moreover, GST activity (Figure 9E) in the Fe group was lower than the control group (*p* = 0.0032). In contrast, the CC and CC + Fe groups demonstrated GST activities comparable to the control, indicating an activity increase relative to the Fe group (*p* = 0.0068 and *p* = 0.0106, respectively). Cell viability, measured through the resazurin reduction assay (Figure 9F), was lower in the Fe group compared to the control, indicating diminished cellular health (*p* < 0.0001). Conversely, the CC and CC + Fe groups exhibited enhanced resazurin reduction capabilities, signifying greater cell viability compared to the Fe group (*p* < 0.0001 for both).

Lastly, mortality rates (Figure 9G) were significantly higher in flies exposed only to Fe compared to the control group (*p* = 0.0061). The group exposed solely to camu-camu showed reduced mortality compared to the Fe group (*p* = 0.0021). Notably, flies exposed to both camu-camu during development and Fe post-hatching (CC + Fe group) also exhibited a reduced mortality rate relative to the Fe-only group (*p* = 0.0442).

## 4. Discussion

Camu-camu is a nutrient-rich fruit from the Amazon, but its consumption is limited due to certain restrictions *in nature.* However, camu-camu powder is now being sold as a food supplement, and its high vitamin C content is appealing. However, we stress the importance of studying the safety of substances that are being sold without any in vivo toxicological reference, even if they are natural products, as everything depends on the concentration consumed. Despite this, camu-camu powder is being used as a food supplement, although there is a lack of established safe doses, not only considering its potential toxic effect but also its impact on different stages of development due to the modulation of oxidative metabolism. Therefore, it is important to evaluate the safety of camu-camu powder on the development of organisms and its impact on neural function in the basal redox state. For this, we used the *D. melanogaster* model organism, which has a fast life cycle and is easy to handle, allowing us to observe developmental changes.

First, we determined the LC_50_ of camu-camu supplementation in second-stage larvae. These larvae are particularly sensitive to toxicological effects, making them ideal for our study. The LC_50_ was estimated to be 4.799 mg/mL. Based on these findings, we selected three non-lethal concentrations (0.1, 0.5, and 1 mg/mL), as well as two sub-lethal concentrations (5 and 10 mg/mL), to evaluate the effects of camu-camu on preimaginal development.

The sub-lethal concentrations (5 and 10 mg/mL) resulted in developmental impairments, including reduced pupation and hatching rates. Second-stage larvae exposed to these concentrations did not complete their development cycle and did not reach the pupal period (indicated by a reduction in pupal formation). Those that did reach the pupal stage did not undergo metamorphosis, did not form, and therefore did not hatch. The larva can repair damage to the imaginal discs in the first two larval stages (first and second stage), but this possibility of regeneration is lost at the end of the third stage [28]. Imaginal discs are aggregates of cells present in larvae that develop during the pupal period, giving rise to parts of the fly’s body and organs. Since exposure to camu-camu began in the second stage, the repair time may have been very short. On the other hand, the damage can be attributed to high levels of vitamin C in camu-camu, as in a study, larvae were unable to develop in high concentrations of ascorbic acid, and the authors attributed this damage to the pro-oxidant effect [29].

Flies exposed to these sub-lethal concentrations during development also showed a reduction in AChE activity. The impact of camu-camu supplementation in the preimaginal period (second-stage larvae) was assessed by measuring AChE activity as a marker of neural function in adult organisms. It is important to note that the evaluation of the impact of camu-camu supplementation in the preimaginal period (second-stage larvae) on markers of neural function in adult organisms was based on the mechanisms of *D. melanogaster* neurogenesis previously described. Neurogenesis in flies involves two phases, embryonic and larval, separated by a period of mitotic quiescence. This result suggests that neurogenesis may have been affected by exposure to sub-lethal concentrations, since neurogenesis in flies occurs mostly (90%) during the larval period, and these neurons are preserved into adulthood [10]. Neural function is closely related to the locomotor capacity of the flies [30]. Although not biochemical, locomotion and climbing behaviors are parameters for assessing neuronal damage [31]. Therefore, flies exposed to sub-lethal doses showed reduced locomotion within the test arena and took longer to climb.

To estimate the impact of antioxidant exposure during the larval stage on the neural function of adult flies, we evaluated biochemical and molecular parameters under the basal redox status to estimate the effect of larval exposure to antioxidant components on adult neural function. The reactive species assay quantifies both reactive oxygen and nitrogen species [21]. We observed that adult flies exposed to sub-lethal concentrations during development produced greater amounts of RS, although there was no change in lipid peroxidation levels. The term ROS refers to species derived from O_2_ that are more reactive than diatomic oxygen, including the superoxide anion and hydroxyl radical, as well as non-radical species such as H_2_O_2_ [32]. Furthermore, the protective effects of ascorbic acid are not related to the restoration of GSH levels in cells induced by oxidative stress [33]. Thus, the decrease in GST activity observed at sub-lethal concentrations may be due to the reduction in GSH levels caused by the increased RS. It is important to note that we used *D. melanogaster* during the larval period when the proteins and antioxidant system are formed. Supplementation with camu-camu powder in sub-lethal doses impairs the activity of antioxidant enzymes. Clinical studies show that excess antioxidant supplements negatively affect the antioxidant system [34]. In the case of CAT activity, it was increased, indicating the presence of high levels of H_2_O_2_ in sub-lethal concentrations, but there was no change in gene expression. In fact, SOD and CAT can be regulated by post-transcriptional mechanisms, even though the expression of genes encoding antioxidant enzymes is normal. The antioxidant enzymes CAT and SOD control the levels of H_2_O_2_ and superoxide anion and can modulate gene expression at specific concentrations [35].

In the case of SOD, sub-lethal concentrations reduce its activity, but superoxide anion dismutation continues at lower levels. However, at a specific concentration of 5 mg/mL, SOD gene expression was induced to increase. In the second stage of our research, we used concentrations of 0.5 mg/mL based on safety data. We chose this concentration because it did not have a harmful effect on the dose–response curve in an organism in a basal redox state. Increased Fe levels in the body lead to oxidative stress through the Fenton and Haber–Weiss reactions, which stimulate the synthesis of superoxide and hydroxyl radicals [36]. Flies in the CC + Fe group maintained RS and lipid peroxidation levels at the control level. Our results resemble those obtained with camu-camu seed extract in rats [37]. Supplementation with camu-camu powder during the preimaginary period maintained SOD and CAT activity similar to the control. Full GST activity after exposure to Fe suggests no glutathione depletion. Therefore, camu-camu powder modulated the flies’ antioxidant system, protecting and ensuring the long-term functioning of antioxidant enzymes. Exposure to Fe compromised neural function, as observed by AChE activity, locomotion, and climbing behavior in the flies. However, preimaginal exposure to camu-camu protected neural function. In a study by Poetini et al. [13], exposure to Fe increased the levels of this metal in the heads of flies, and hesperidin, when offered concomitantly, regulated AChE activity through a chelating action. Unlike these studies, exposure to camu-camu powder did not occur concomitantly with the stressor, ruling out a chelating action and also demonstrating the ability to positively modulate the redox state in the long term, strengthening the organism against oxidative stress, thus preserving AChE activity and fly locomotion. Fe-induced oxidative damage resulted in increased fly mortality within 24 h. Camu-camu protected and strengthened the flies’ organisms against oxidative damage, preserving the mortality rate of flies subsequently exposed to Fe, similar to the control group. Our findings align with research on natural antioxidants such as *Ilex paraguariensis* powder, which, when combined with methylmalonic acid, maintained mortality rates at a controlled level [38]. Our study highlights non-concurrent exposure to the stressor (Fe) as a differentiator. Organisms exposed during development to camu-camu and as adults to oxidative insults presented a protective effect, strongly modulating the fly’s antioxidant defenses. This effect can be attributed to the levels of vitamin C (at a safe concentration) present in the powder. Vitamin C is highly susceptible to thermal and oxidative degradation, making it challenging to maintain its physiological value over time. Nonetheless, its lipophilic portions can be structurally modified to form various ascorbic acid derivatives, which offer greater thermal and oxidative stability [39]. These derivatives can effectively address oxidative damage in biological molecules, particularly nucleic acids and lipid membranes [40]. Furthermore, pretreatment with vitamin C can help regulate oxidative stress through epigenetic mechanisms, bolstering the organism’s redox state [41]. The observed effect in this study demonstrates how vitamin C from camu-camu powder exerts a modulating action on the flies’ redox state. This effect is achieved through a free radical scavenging mechanism, in which vitamin C donates electrons, regenerates other antioxidants, neutralizes hydroxyl radicals, and protects against damage from singlet oxygen. These mechanisms help maintain cell integrity and protect the organism against oxidative stress and its effects, particularly at a neurological level, as shown by AChE activity. It is worth noting that all analyses revealed a perceptible effect exerted by camu-camu powder. In the group that used the isolated powder, effective redox regulation was observed. The administration of camu-camu powder during the preimaginal period was observed to have long-term effects, making it possible to observe greater resistance to oxidative stress induced in flies. This finding indicates that pretreatment (supplementation) can act on oxidative stress, strengthening the body’s redox state. Despite the robust data presented, an important limitation of this study is the lack of detail on the composition of camu-camu powder. Given its strong antioxidant effect, it would be valuable to identify the compounds present. In our study, we used commercial camu-camu powder and attributed our results to vitamin C based on the nutritional table provided by the manufacturer. It is worth noting that vitamin C comprises 70% of the total antioxidant capacity of camu-camu [42]. In 5 g of camu-camu powder, there is 682 mg of vitamin C, and while few studies have reported the presence of other compounds in dry camu-camu, one study found, in 100 g (dry powder), 42 mg of quercetin, 0.4–2.5 mg of kaempferol, and 0.13 mg of rutin [43,44,45,46,47]. Including an HPLC analysis in future studies would enhance our understanding of the composition of camu-camu powder and further clarify the benefits of using this supplement.

## 5. Conclusions

Our study on supplementation with camu-camu powder during the preimaginal development stages of *Drosophila melanogaster* highlights its complex impact on redox state organisms. We observed that sub-lethal concentrations of camu-camu induced a pro-oxidant effect beginning from the larval stage. This was evidenced by decreased preimaginal viability, inhibited fly hatching, and extended into adulthood, as revealed through various tests and analyses.

Following the dose–response curve, a concentration of 0.5 mg/mL (roughly ten times lower than the LC_50_) was used in tests to investigate the antioxidant protective effect of camu-camu against oxidative stress induced by exposure to Fe. Indeed, supplementation with camu-camu powder at a non-lethal concentration demonstrated that it modulated and strengthened the antioxidant system against oxidative stress in the long term, developed during the larval period. These insights are pivotal in evaluating the safety and efficacy of camu-camu as a commercial supplement and highlight the importance of dose-dependent effects and the necessity for comprehensive studies to establish safe and effective supplementation guidelines.

## Figures and Tables

**Table 1:** Nutritional characteristics of Camu-camu organic commercial powder supplement.

Macro and Micronutrients	Concentration at 5 g/Product
Total carbohydrate	4 g
Vitamin C	682 mg
Potassium	39 mg
Sodium	15 mg
Calcium	4 mg
Total fat	0 g
Protein	0 g
Iron	0 g

**Table 2:** Gene primers list.

Genes	Primers	
	Sense	Antisense
GPDH *Gene ID: 33824*	ATGGAGATGATTCGCTTCT	GCTCCTCAATGGTTTTTCCA
SOD *Gene ID: 36878*	ACCGCACTTCAATCCGTAG	AGTCGGTGATGTTGACCTTG
CAT *Gene ID: 40048*	ACCAGGGCATCAAGAATCG	AACTTCTTGGCCTGCTCGTA

## Data Availability

The original contributions presented in the study are included in the article, further inquiries can be directed to the corresponding author.
